# Potential delay in the diagnosis of vulvar cancer and associated risk factors in women treated in German gynecological practices

**DOI:** 10.18632/oncotarget.23848

**Published:** 2018-01-03

**Authors:** Jennifer Muigai, Louis Jacob, Konstantinos Dinas, Karel Kostev, Matthias Kalder

**Affiliations:** ^1^ Department of Gynecology and Obstetrics, Philipps-University Marburg, Marburg, Germany; ^2^ Faculty of Medicine, University of Paris 5, Paris, France; ^3^ Aristotle University of Thessaloniki, Faculty of Medicine, Thessaloníki, Greece; ^4^ Epidemiology, QuintilesIMS, Frankfurt am Main, Germany

**Keywords:** vulvar cancer, delay, risk factors, vulva infection, vulva inflammation

## Abstract

The goal of this study was to estimate a potential delay in the diagnosis of vulvar cancer and identify associated risk factors in women treated in gynecological practices in Germany. The current study sample included 1,652 women from 218 gynecological practices who received an initial diagnosis of vulvar cancer (ICD 10: C51) between January 2000 and December 2015 (index date). After applying several exclusion criteria, 505 non-cancer controls were matched (1:1) to 505 vulvar cancer cases based on age, health insurance status, and index date. The primary outcome was the delay in vulvar cancer diagnosis in women who had received an initial diagnosis of diseases of Bartholin's gland, inflammation of vagina and vulva, or other specified noninflammatory disorders of vulva, including atrophy, hypertrophy, and cyst. A logistic regression model was used to estimate the association between vulvar cancer and pre-defined diagnoses. The mean age was 60.8 years (SD = 15.6 years), and 4.8% of women had private health insurance coverage. Inflammation of vagina and vulva was diagnosed 328 days (SD = 95 days) prior to the detection of vulvar cancer. This delay was 186 days (SD = 196 days) in patients affected by diseases of Bartholin’s gland and 300 days (SD = 116 days) in those with other specified noninflammatory disorders of vulva including atrophy, hypertrophy, and cyst. The risk of vulvar cancer was positively associated with inflammation of vagina and vulva (OR = 2.28) and other specified noninflammatory disorders of vulva (OR = 5.39). The mean potential delay of vulvar cancer diagnosis ranged from 186 to 328 days.

## INTRODUCTION

Vulvar cancer accounts for an important share of all gynecological malignancies [1]. In Germany, 4% of gynecological cancers involve a cancer of the vulva [2]. Approximately 3,200 cases were reported in this country in 2012, and this number is supposed to have increased to 4,400 cases in 2016 [3]. Therefore, vulvar cancer has an increasing impact on health and its associated economy in Germany.

A delay in the diagnosis of vulvar cancer has been reported in the literature and seems to result from a lack of specificity of symptoms at the early stages of the disease. Such delay has been found to vary from 6 to 22 months in studies performed in recent decades [4–8]. When symptoms are present, they involve skin thickening, pruritus, pain, burning sensations, bleeding or discharge unrelated to the normal menstrual cycle, open sores, and lumps [4, 9]. Pruritus, a symptom frequently associated with most vulva diseases, is particularly common in vulva dystrophy (e.g., lichen sclerosus and eczema), menopause, and bacterial or fungal infections. Contact dermatitis is also frequently found in Western countries due to an increased use of topical products potentially harmful to the vulva [10]. Interestingly, a German analysis including 208 patients affected by vulva disorders further showed that 63.9% of the population was diagnosed with inflammatory diseases [11]. Another condition that shares similar symptoms with vulvar cancer is postmenopausal vulvovaginal atrophy (VVA), a disorder associated with a deficiency in estrogen. Corroborating recent results published by the North America Menopause Society [12], the prevalence of VVA symptoms was estimated at 40% in Germany, with half of the afflicted women describing their symptoms as either moderate or severe [13]. Finally, vulvovaginal candidiasis, a common vulva disorder found in 30-50% of the population, may also lead to a delay of vulvar cancer diagnosis [14].

Since multiple symptoms are associated with vulvar cancer and a wide range of benign vulva disorders, vulvar cancer remains difficult to diagnose. Although several works have already estimated the delay in the diagnosis of vulvar cancer [4–8], most of them were conducted in the late 1990s, and none were performed in Germany. Therefore, the goal of the present study was to estimate the delay in the diagnosis of vulvar cancer and to identify potential risk factors in women treated in German gynecological practices.

## MATERIALS AND METHODS

### Database

This retrospective study is based on data from the Disease Analyzer database (QuintilesIMS), which compiles demographic, clinical, and pharmaceutical data obtained in an anonymous format from computer systems used in clinical practices [15]. The quality and exactness of the data (e.g., diagnoses and drug prescriptions) are regularly assessed by QuintilesIMS. Using prescription statistics for several drugs and age groups for several diagnoses, the Disease Analyzer database was found to be a representative database of clinical practices in Germany [15]. Finally, several studies focusing on female cancers and using the same database have already been published [16, 17].

### Study population

**Figure 1:**
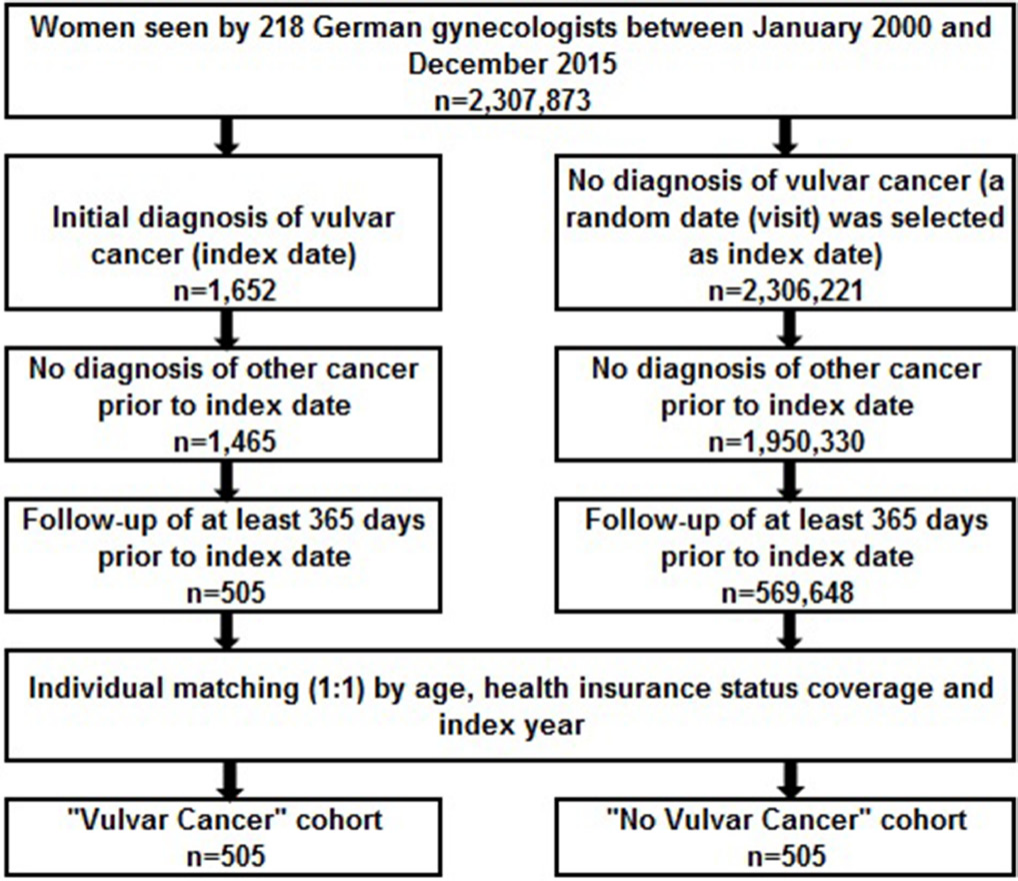
Flow chart of women included in the present German study (Disease Analyzer database, QuintilesIMS)

Figure 1 shows the patient selection process. The current study sample included 1,652 women from 218 gynecological practices who received an initial diagnosis of vulvar cancer (ICD 10: C51) between January 2000 and December 2015 (index date) and no prior cancer diagnosis. Patients were excluded if they had been diagnosed with any other cancer (C00-99) prior to vulvar cancer diagnosis. In order to guarantee the accuracy of diagnoses and enable the analysis of pre-diagnoses, patients with an observation period prior to their index date of less than 365 days were excluded. After applying these exclusion criteria, 505 vulvar cancer patients were included in the study. During the index period, 1,950,330 women observed in these 218 gynecological practices had no cancer diagnosis. After applying similar exclusion criteria, 569,648 non-cancer patients were eligible to be included in the present investigation. Finally, 505 non-cancer controls were matched (1:1) to vulvar cancer cases based on age (in years), health insurance status (private versus statutory health insurance coverage), and index date. In the control group, a random date (medical visit) was selected and designated the index date, ensuring an observation period prior to the index date of at least 365 days. When more than one matched individual was available in the control group for one vulvar cancer patient in the case group, one of these controls was randomly selected.

### Study outcome

The primary outcome was the potential delay in vulvar cancer diagnosis in women who had received an initial diagnosis of diseases of Bartholin’s gland (N75), inflammation of vagina and vulva (N76), or other specified noninflammatory disorders of vulva including atrophy, hypertrophy, and cyst (N90.5-90.9) in the year prior to the vulvar cancer diagnosis.

### Statistical methods

Descriptive analyses were obtained for age, index year, and health insurance coverage using paired *t*-tests, Wilcoxon-tests for paired samples or McNemar’s tests. A logistic regression model was used to estimate the association between vulvar cancer and pre-defined diagnoses (diseases of Bartholin’s gland, inflammation of vagina and vulva, or other specified noninflammatory disorders of vulva including atrophy, hypertrophy, and cyst). The following diagnoses known to be associated with a higher risk of vulvar cancer were included as co-variables: vulva dysplasia (N90.0–90.3), leukoplakia of vulva (N90.4) and benign neoplasm of vagina (D28.1). *P*-values < 0.05 were considered statistically significant. The analyses were carried out using SAS version 9.3.

## RESULTS

Baseline characteristics of the women included in the study are displayed in Table 1. After individual matching, the mean age was 60.8 years (SD = 15.6 years), and 4.8% of women had private health insurance coverage. Inflammation of vagina and vulva was diagnosed 328 days (SD = 95 days) before vulvar cancer was detected. This delay was 186 days (SD = 196 days) in patients affected by diseases of Bartholin’s gland and 300 days (SD = 116 days) in those with other specified noninflammatory disorders of vulva including atrophy, hypertrophy, and cyst. Table 2 shows the results of the multivariate logistic regression model performed in the present work. The risk of vulvar cancer was positively associated with inflammation of vagina and vulva (OR = 2.28) and other specified noninflammatory disorders of vulva including atrophy, hypertrophy, and cyst (OR = 5.39).

**Table 1:** Characteristics of patients with vulvar cancer and controls treated in gynecological practices in Germany

	Prior to 1:1 matching			After 1:1 matching^*^		
Variables	Vulvar cancer patients	Controls	*P*-value^*^	Vulvar cancer patients	Controls	*P*-value^*^
N	505	569,648		505	505	
Variables used for matched-pairs						
Age (years)	60.8 (15.6)	40.8 (16.5)	< 0.001	60.8 (15.6)	60.8 (15.6)	1.000
Age ≤ 40	10.3	55.4	< 0.001	10.3	10.3	1.000
Age 41–50	17.5	17.6	0.956	17.5	17.5	1.000
Age 51–60	19.5	12.1	< 0.001	19.5	19.5	1.000
Age 61–70	19.3	8.5	< 0.001	19.3	19.3	1.000
Age > 70	33.4	6.4	< 0.001	33.4	33.4	1.000
Private health insurance coverage	4.8	9.3	< 0.001	4.8	4.8	1.000
Index year						
2000–2005	7.2	16.4	1.000	7.2	7.2	1.000
2006–2010	21.4	22.4	0.625	21.4	21.4	1.000
2011–2015	71.4	61.2	< 0.001	71.4	71.4	1.000
Other variables^**^						
Vulva dysplasia (ICD10: N90.0-90.3)	8.3	0.1	< 0.001	8.3	0.2	< 0.001
Leukoplakia of vulva (ICD10: N90.4)	14.5	0.6	< 0.001	14.5	1.4	< 0.001
Benign neoplasm of vagina (ICD10: D28.1)	1.2	0.0	< 0.001	1.2	0.0	0.014

Matching based on age (in years), health insurance status (private versus statutory health insurance coverage) and index date.

^*^p-value < 0.05 (vulvar cancer vs. controls): paired t-tests, Wilcoxon-tests for paired samples, or McNemar´s tests.

^**^Only diagnoses documented within 12 months prior to the index date were considered.

**Table 2:** Association between vulvar cancer and predefined diagnoses

Patient cohort	% of vulvar cancer patients	% of non-cancer patients	Odds ratio (95%CI)^*^	*P*-value
Diseases of Bartholin’s gland (N75)	0.6	0.6	1.25 (0.25–6.21)	0.787
Inflammation of vagina and vulva (N76)	48.1	27.1	2.28 (1.73–3.24)	< 0.001
Vulvovaginal ulceration and inflammation in diseases classified elsewhere (N77)	0	0	NA	NA
Other specified noninflammatory disorders of vulva including atrophy, hypertrophy, and cyst) (N90.5–90.9)	5.0	0.8	5.39 (1.81–16.06)	0.003
At least one of the above diagnoses	51.3	28.1	2.40 (1.83–3.14)	< 0.001

Results of the logistic regression model analysis.

^*^Adjusted for vulva dysplasia, leukoplakia of vulva, and benign neoplasm of vagina.

## DISCUSSION

The present study comprising 505 women with vulvar cancer and 505 women without vulvar cancer showed that the potential delay in the diagnosis of cancer ranged from 186 days in patients receiving a first diagnosis of diseases of Bartholin’s gland to 328 days in those receiving a first diagnosis of inflammation of vagina and vulva.

Few authors have studied the duration between first symptoms and final diagnosis in women with vulvar cancer. In 1997, Rosén and Malmström discovered in 328 patients with histologically confirmed primary invasive vulvar cancer that they experienced symptoms during an average of 16.3 months before diagnosis was made [4]. The most frequent symptoms were pruritus, smarting pain, and vulva tumor. Stages I, II, III, and IV accounted for 35%, 37%, 15%, and 13% of individuals with squamous cell carcinomas, respectively. Tumor stage was found to be negatively associated with survival. Later, a UK study including 506 individuals with carcinoma of the vulva estimated that diagnosis was delayed by more than a year in approximately one out of five women [18]. The number of symptoms and their duration were further found to have no significant impact on survival.

In 1999, Jones and Joura evaluated clinical events preceding the diagnosis of squamous cell carcinoma of the vulva [5] and showed in this New Zealand study including 102 women that 88% of them experienced vulva symptoms for more than six months and 28% of them for more than five years. Interestingly, abnormal skin adjacent to the tumor was found in 85% of the population, and 31% of women had at least 3 medical consultations for vulva symptoms prior to the diagnosis of vulvar cancer. Finally, 25% of patients underwent vulva biopsy, and these patients were more likely to present stage 1 disease than those without vulva biopsy. Finally, Voliovitch et al. aimed to analyze the clinical features of Israeli patients affected by squamous cell carcinoma of the vulva [19]. The authors discovered in 150 women that pruritus was the most frequent symptom. Corroborating previous studies, a considerable time lag was observed from onset of symptoms to diagnosis.

More recently, in 2009, Lanneau and colleagues discovered in 56 patients under the age of 45 that 47% of this population presented symptoms for more than 12 months prior to the diagnosis of vulvar cancer [7]. It was further estimated that the delay between first symptoms and final diagnosis correlated with neither stage nor positive lymph nodes. Two years later, a Danish study including 161 women with gynecological cancers who had responded to four different questionnaires found that median total delay was 101 days [8]. Gynecological cancers included ovarian, endometrial, cervical, and vulvar cancers. Approximately 10% of the population exhibited a delay of at least 436 days. Vulvar cancer was further associated with the longest delay, while ovarian cancer was associated with the shortest delay. In this analysis, more than one third of individuals consulted their general practitioner for trivial symptoms, not for alarming symptoms. Moreover, physicians were less likely to perform gynecological examinations when vaginal bleeding was absent, although the length of delay was shortened when such an examination was conducted.

In line with these various works, the present German study comprising 1,010 women included between 2000 and 2015 showed that a potential delay in the diagnosis ranged from around six to 11 months. An interesting finding of this retrospective analysis is that such delay varied with the initial diagnosis given by gynecologists, with inflammation of vagina and vulva being associated with the longest delay and diseases of Bartholin’s gland with the shortest delay. Although diseases of Bartholin’s gland were diagnosed in less than 1% of the population, potentially causing an inaccurate estimation of the associated delay, such difference in delays may be explained by a difference in the symptomatology presented by women with these various benign vulva disorders. As no evidence in the literature has yet supported this hypothesis, such a finding must be interpreted with great caution.

A delay in vulvar cancer diagnosis may also be caused by other reasons, such as physical disability of some patients, fear of special exams, or reluctance to get gynecological exams. Patients may also fail to recognize cancer symptoms or act on them. Furthermore, diagnostic delay may also occur if several invasive procedures are needed to confirm the diagnosis [20].

Retrospective primary care database analyses are generally limited by the validity and completeness of the data on which they are based. The present study was subject to several limitations, such as the assessment of vulvar cancer and co-morbidities, which relied solely on ICD codes entered by gynecologists. Furthermore, data pertaining to socioeconomic status (e.g., education and income) and lifestyle-related risk factors (e.g., physical activity) were also lacking. Furthermore, no documentation is provided on how the cancer diagnoses were made; however, in Germany, cancer diagnoses are usually confirmed by hospitals. Finally, the database is representative of resident physicians and does not include data from hospitals.

The study also had several strengths. More than 1,000 women from several gynecological practices were included. Another strength was the use of real-time treatment data in gynecological practices where diagnoses were continuously documented, allowing for unbiased exposure assessment (no recall bias).

Overall, potential mean delay of vulvar cancer diagnosis ranged from 186 to 328 days. Further research is needed to gain a better understanding of the impact of benign vulva disorders on this delay. Such research could lead to an improvement in the management and treatment of women newly diagnosed with vulvar cancer. Moreover, enabling access to specialist expertise (like university clinics) through prompt referrals should help prevent delays in cancer diagnosis.
